# Precise extraction of impacted supernumerary tooth in the maxillary anterior region with a digital guide plate: A case report

**DOI:** 10.1097/MD.0000000000029275

**Published:** 2022-05-27

**Authors:** Fangyong Zhu, Deqiang Hou, Chen Zhou, Zhifei Chen, Yannan Cao, Lian Ji, Jianming Zou, Yanhua Xu

**Affiliations:** a Department of Stomatology, Affiliated Hospital of Jiangnan University, Wuxi, China; b Department of Orthodontics, Affiliated Stomatology Hospital of Kunming Medical University, Kunming, China.

**Keywords:** cone beam computed tomography, dental model, digital guide plate, three-dimensional

## Abstract

**Rationale:**

Removal of impacted supernumerary teeth requires precision and accuracy to prevent iatrogenic injury to important anatomical structures during dental surgery and to improve postoperative healing.

**Patient concerns:**

A 12-year-old girl visited our department for the assessment and management of her deviated front teeth.

**Diagnosis:**

Impacted supernumerary tooth extraction in the maxillary anterior region.

**Interventions:**

The digital guide plate was fabricated after the integration of cone beam computed tomography data with that obtained from scanning the patient's dental model. Impacted supernumerary tooth extraction was performed.

**Outcomes:**

The use of the digital guide plate and planting instruments made the removal of the impacted supernumerary tooth less invasive, faster, and more accurate, whereas the wound was smaller, and the patient experience more comfortable.

**Lessons:**

Combining the digital guide plate with planting instruments offers a useful aid for the removal of impacted supernumerary teeth among the maxillary anterior region and is, thus, worth promoting.

## 1 Introduction

Maxillary supernumerary teeth are commonly seen among children in the mixed dentition stage.^[1]^ Some maxillary supernumerary teeth are completely impacted and cannot be easily detected unless when complications occur, such as failure of eruption, diastemas, dental caries, and other lesions, which necessitate their removal. Radiography is the mainstay of supernumerary tooth detection.^[2]^ Before cone beam computed tomography (CBCT), clinicians would decipher the location of the embedded teeth, their relationship with the adjacent teeth, and the important anatomical structures surrounding them two-dimensionally via orthopantomography, occlusal films, or periapical films from different angles.^[3]^ The emergence of CBCT and three-dimensional (3D) reconstruction renders such information more comprehensive and accurate, but these techniques still have limitations.^[3]^ The transfer of information obtained through these techniques to the operative site still remain a challenge. This uniqueness case report presents the use of a digital guide plate enabling the surgeons perform minimally invasive tooth removal comprehensively and accurately, which is helpful and effective, thus enabling the surgeons perform minimally invasive tooth removal comprehensively and accurately.

## 2 Case presentation

### 2.1 Patient information

Chief complaint: a 12-year-old girl visited our department for the assessment and management of her deviated front teeth. This patient said the embedded teeth were often painful, especially when eating. The embedded teeth were required to be removed. The patient did not have significant past medical history. During routine panoramic examination of the oral cavity, an impacted supernumerary tooth was found close to the roots of her upper central incisors. The patient's parents agreed to remove this impacted tooth.

### 2.2 Presurgical preparation

**Figure 1:**
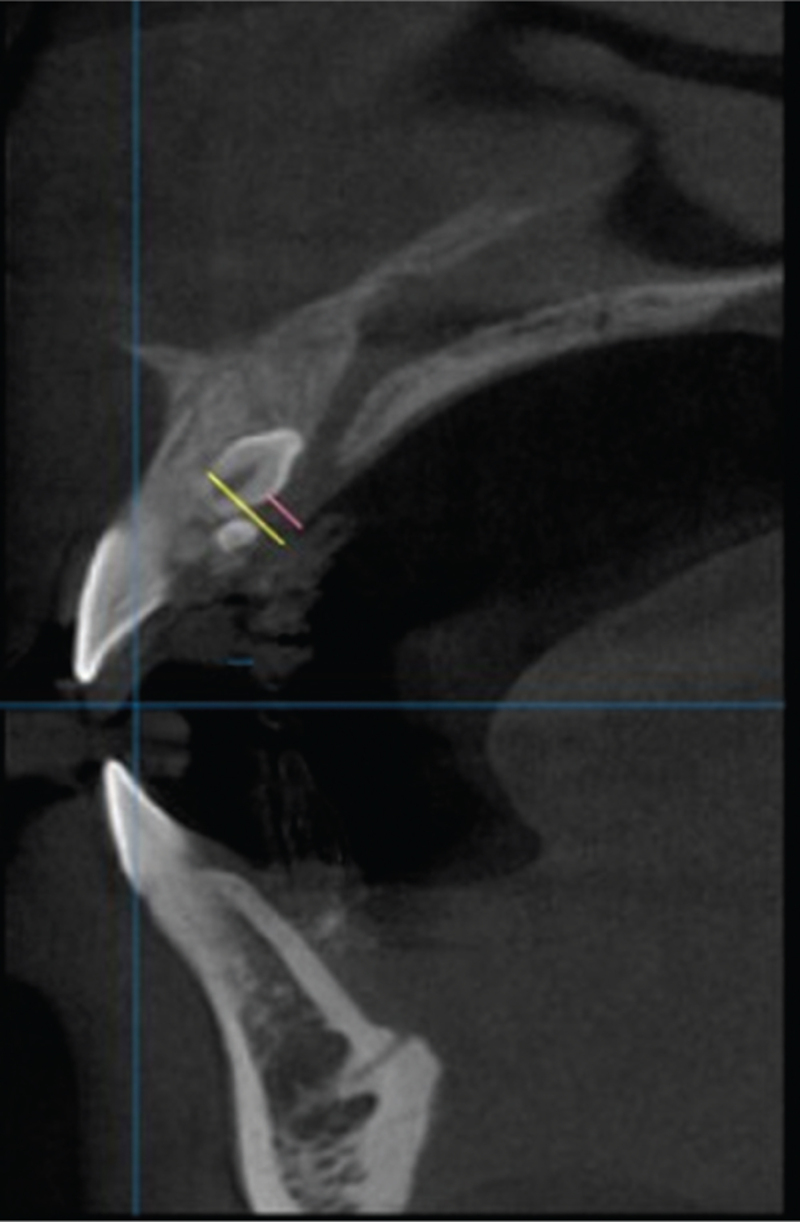
Cone beam computed tomography image of the patient's mouth in sagittal view. The yellow line marks the distance from the supernumerary tooth's labial margin to the palatal mucosa (L1). The yellow line marks the distance from its palatal margin to the mucosa (L2).

The patient's maxillary impression was made, and a dental plaster model was fabricated. It was scanned and the scan was exported in STereoLithography format. The patient then underwent CBCT and communication (Digital Imaging and Communications in Medicine) format. A digital 3D dental model was reconstructed. A sagittal section on the CBCT revealed the impacted supernumerary tooth to be on the palatal side between the roots of the 2 central incisors. The distance from the supernumerary tooth's labial margin to the palatal mucosa (L1) and the distance from its palatal margin to the mucosa (L2) were measured in the sagittal plane (Fig. 1). The STereoLithography and Digital Imaging and Communications in Medicine files were then integrated using the 3SHAPE software.

The digital guide plate was designed after data integration. The guide plate's edge was located 5 mm distal to the tooth. The other edges were adjusted for the entry of scopes, for surgical convenience, and to avoid iatrogenic injury to important anatomical structures. The digital plate was exported in STereoLithography format, printed through stereolithography 3D printing, assembled with a guide ring, tried on the plaster model for further modifications, and then disinfected and tried on the patient. During the trial, the clinicians observed the plate's stability and retention, and the ill-fitting parts were modified accordingly, so as to achieve an accurate fit.

Surgical instruments included a wide-angle high-speed turbine, a round bur for bone removal, and a fissure bur to section the tooth. The lengths of the round bur and fissure bur were selected according to the L1 and L2 measurements. A compatible stop piece was selected and placed on its appropriate position – on root canal file in this case – according to the height of the guide ring. The guide plate and instruments were all sanitized and disinfected.

### 2.3 Dental surgery

**Figure 2:**
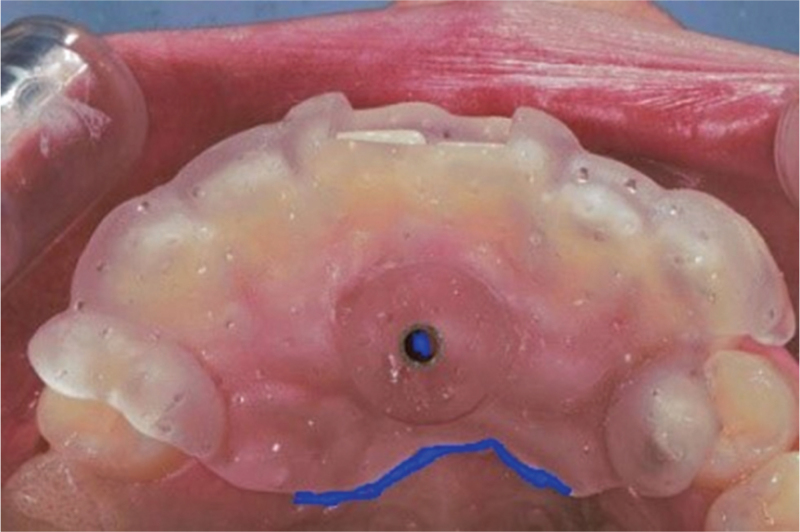
Placement of the digital guide plate in the patient's mouth, with the plate's edge and the supernumerary tooth's center indicated.

**Figure 3:**
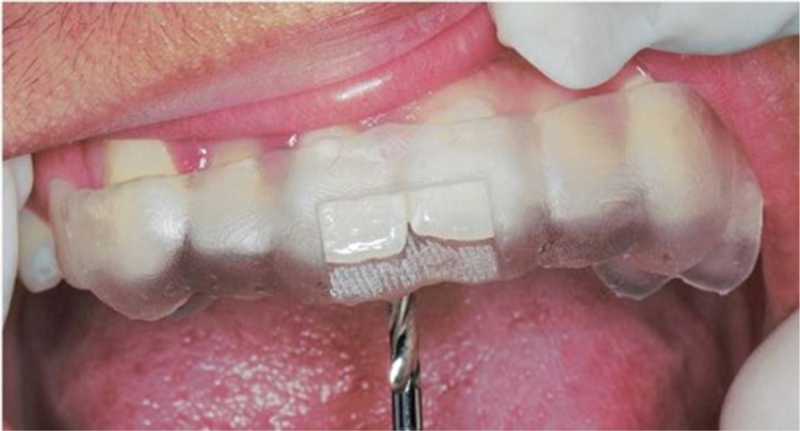
The guide ring was used to determine the depth for the fissure bur to remove bones from the surface of the hard palate.

**Figure 4:**
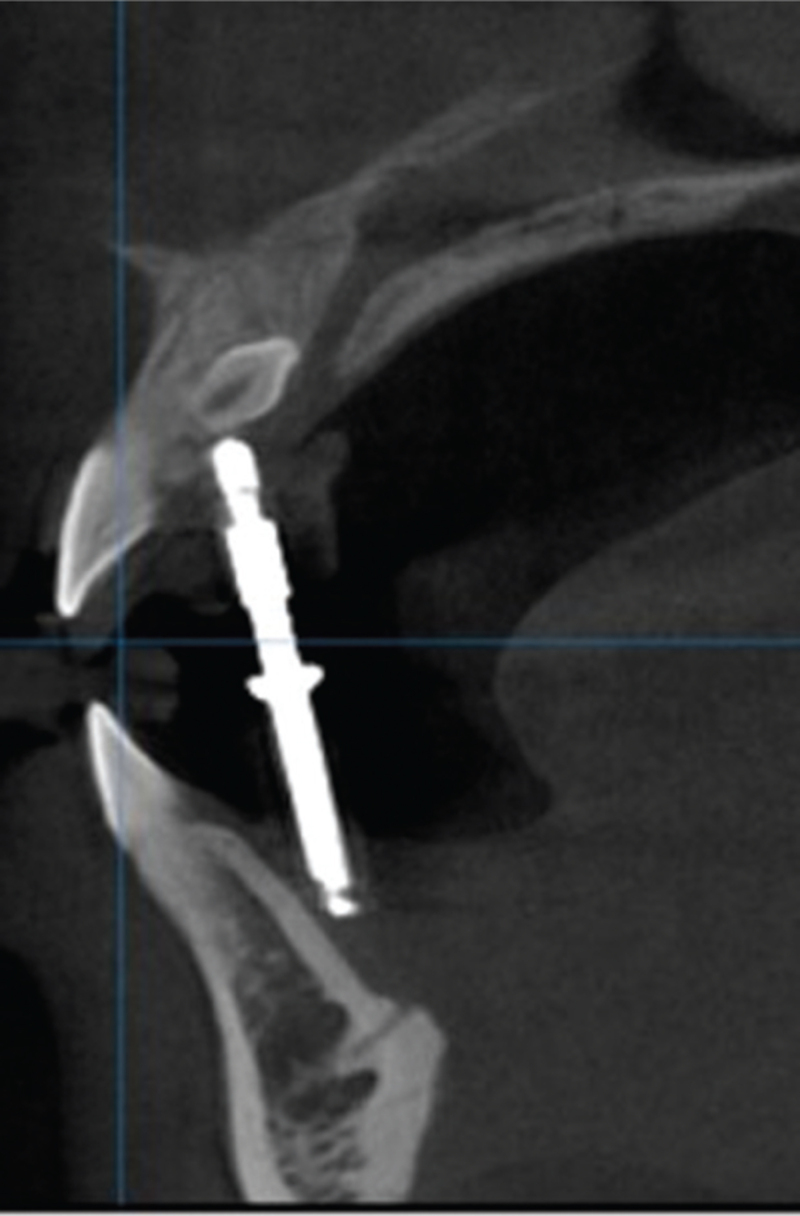
The guide ring was used to determine the depth of the fissure bur, in order to remove bone, as seen under cone beam computed tomography.

The operative region was disinfected, and local anesthesia (Primacaine) was administered. The mucosa delineated by the guide ring, which represented the center of the impacted tooth, was marked with a methylrosanilinium chloride swab. A line was drawn along the palatal edge of the guide plate, which represented the extent of the operative region (Fig. 2). The incisions for the flap were determined by the palatal extent of the guide plate and the inciso-apical length of the impacted tooth, as determined by the CBCT. Laterally, the incisions were so planned that the point indicated by the guide ring was midway between the left and right lateral incisions. The incisions were made after removal of the surgical guide plate, and the mucoperiosteal flap was elevated. Bleeding was controlled and the guide plate was reinserted. The stopper on the round bur's shank was adjusted to account for L2 and the thickness of the guide ring to determine the depth for the high-speed turbine and round bur for palatal bone removal (Fig. 3). After adjustment of the stopper, the guide plate was removed and the round bur used to gradually remove the bone overlying the impacted tooth in circular motion, centered on the depth-fixed hole, with the bur direction being continuously adjusted to avoid any unnecessary bone removal. After gradually exposing the impacted tooth, the fissure bur was used to section it for removal. The depth was determined intraorally by advancing the fissure bur through the guide ring until the stop piece reached the surface of the guide plate. The guide plate was then removed, the tooth gradually sectioned, and pulled it out in pieces (Fig. 4). Finally, the post-extraction defect was cleaned and rinsed with normal saline, the bleeding controlled, and the flap sutured.

The surgery lasted approximately 30 minutes. The patient cooperated well and did not experience obvious discomfort. Postoperatively, the patient was prescribed anti-inflammatory drugs and analgesics and was advised to gargle with mouthwash the next day. In the subsequent visit, after 24 hours, the operative region was mildly edematous and exhibited some inflammation. After 3 days, the swelling had reduced. The sutures were removed after a week. Six months after surgery, this patient had a good treatment effect, and the wound healed well without complication, including no damage of adjacent teeth or nerves.

## 3 Discussion

Supernumerary teeth refer to the extra teeth in the jaw. This type of odontogenic dysplasia is common in clinical practice. It has been reported that the incidence among Caucasians is about 0.1% to 3.8%, while that among Mongolians is 3%. The incidence among males is higher than that in females.^[4]^ There is a high incidence of supernumerary teeth in the anterior maxilla; some of which erupt and some of which are completely impacted. Supernumerary teeth embedded in the anterior maxillary area often cause sequelae, such as maxillary incisor diastemas, failure of eruption, permanent teeth displacement, dentition crowding, root resorption, and dental cysts; hence, they need to be removed.

CBCT may be used to aid in the extraction of impacted supernumerary teeth. Compared with conventional radiographic imaging studies, CBCT can provide accurate and scientific imaging data to help in the diagnosis and provide a more reliable basis for treatment.^[5]^ It can help determine the impacted tooth's depth in the jaw, its labial and palatal position, direction of impaction, and the inclination angle between the impaction and the sagittal plane more accurately; this can help in determining the surgical approach.^[6,7]^ However, CBCT cannot accurately transmit the information related to the impacted supernumerary teeth in the mouth, which reduces the help it offers in the extraction of the impacted teeth to some extent as it makes the scope of operation relatively unclear.

The promotion of minimally invasive tooth extraction and painless technology has brought Chinese alveolar surgery almost at par with advanced international standards. Minimally invasive surgery is the pursuit of all dentists.^[8,9]^ Techniques like the surgical guide plate, 45°-angle turbine, ultrasonic bone knife, surgical navigation assistance system, and minimally invasive tooth extraction instrument significantly reduce the difficulty of complicated tooth extraction.^[10–13]^ The use of a digital guide plate in this case made the extraction of the impacted supernumerary tooth in the anterior maxillary region minimally invasive and accurate. First, the digital guide plate supplemented information from CBCT, which provided accurate information, such as the location of the tooth and its relationship with the adjacent teeth within the mouth. It also helped in the prevention of iatrogenic injury to important anatomical structures. Second, a guide ring was added to the digital guide plate to direct both the course of the drilling needle and the high-speed turbine in grinding the bone plate and during tooth separation. It was able to prevent the displacement of the drilling needle sideways that could have led to grinding of the surgical guide plate or adjacent tissues. Third, the digital guide plate's edge was used as the reference line for the surgical area's edge, which led to better and more accurate control of the surgical incision and the flap. Fourth, the digital guide plate was processed in vitro, which effectively reduced the operating time. Finally, the guide plate was small, comfortable, and, hence, acceptable to the patients, especially children.

To conclude, the best personalized treatment is based on the patient's age, economic conditions, expected goals, and combined with new technology with the need for long-term follow-up.

## Acknowledgments

We thank Chen Zhou, Zhifei Chen, Yannan Cao, and Lian Ji for their help in the treatment of this patient.

## Author contributions

**Conceptualization:** Yanhua Xu.

**Data curation:** Yanhua Xu.

**Formal analysis:** Fangyong Zhu.

**Funding acquisition:** Fangyong Zhu.

**Investigation:** Deqiang Hou.

**Methodology:** Deqiang Hou.

**Project administration:** Chen Zhou.

**Resources:** Chen Zhou.

**Supervision:** Zhifei Chen.

**Validation:** Zhifei Chen.

**Visualization:** Yannan Cao.

**Writing – original draft:** Yannan Cao.

**Writing – review & editing:** Jianming Zou, Jianming Zou.
